# Acute Endovascular Treatment of Patients With Ischemic Stroke From Intracranial Large Vessel Occlusion and Extracranial Carotid Dissection

**DOI:** 10.3389/fneur.2019.00102

**Published:** 2019-02-19

**Authors:** Kars C. J. Compagne, R. B. Goldhoorn, Maarten Uyttenboogaart, Robert J. van Oostenbrugge, Wim H. van Zwam, Pieter J. van Doormaal, Diederik W. J. Dippel, Aad van der Lugt, Bart J. Emmer, Adriaan C. G. M. van Es, the MR CLEAN investigators

**Affiliations:** ^1^Department of Radiology and Nuclear Medicine, Erasmus MC, University Medical Center, Rotterdam, Netherlands; ^2^Department of Neurology, Erasmus MC, University Medical Center, Rotterdam, Netherlands; ^3^Department of Neurology, Maastricht University Medical Center, Maastricht, Netherlands; ^4^Department of Neurology, University Medical Center Groningen, University of Groningen, Groningen, Netherlands; ^5^Department of Radiology, University Medical Center Groningen, University of Groningen, Groningen, Netherlands; ^6^Cardiovascular Research Institute Maastricht, Maastricht, Netherlands; ^7^Department of Radiology, Maastricht University Medical Center, Maastricht, Netherlands; ^8^Department of Radiology, Amsterdam University Medical Center, Amsterdam, Netherlands

**Keywords:** ischemic stroke, carotid dissection, endovascular treatment, tandem lesion, thrombectomy

## Abstract

**Introduction:** Carotid artery dissection (CAD) and atherosclerotic carotid artery occlusion (ACAO) are major causes of a tandem occlusion in patients with intracranial large vessel occlusion (LVO). Presence of tandem occlusions may hamper intracranial access and potentially increases the risk of procedural complications of endovascular treatment (EVT). Our aim was to assess neurological, functional and technical outcome and complications of EVT for intracranial LVO in patients with CAD in comparison to patients with ACAO and to patients without CAD or ACAO.

**Methods:** We analyzed data of the MR CLEAN trial intervention arm and MR CLEAN Registry, acquired in 16 Dutch EVT-centers. Primary outcome was the change in stroke severity by comparing the National Institute of Health Stroke Scale (NIHSS) score at 24–48 h after treatment vs. baseline. Secondary outcomes included reperfusion rate and symptomatic intracranial hemorrhage (sICH). We compared outcomes and complications between patients with CAD vs. patients with ACAO and patients without CAD or ACAO.

**Results:** In total, we identified 74 (4.7%) patients with CAD, 92 (5.9%) patients with ACAO and 1398 (89.4%) patients without CAD or ACAO. Neurological improvement at short-term after EVT in patients with CAD was significantly better compared to ACAO (resp. mean −5 vs. mean −1 NIHSS point; *p* = 0.03) and did not differ compared to patients without CAD or ACAO (−4 NIHSS points; *p* = 0.62). Rates of successful reperfusion in patients with CAD (47%) was comparable to patients with ACAO (47%; *p* = 1.00), but was less often achieved compared to patients without CAD or ACAO (58%; *p* = 0.08). Occurrence of sICH did not differ significantly between CAD patients (5%) and ACAO (11%; *p* = 0.33) or without CAD/ACAO (6%; *p* = 1.00).

**Conclusion:** EVT in patients with intracranial LVO due to CAD results in neurological improvement comparable to patients without tandem occlusions. Therefore, carotid artery dissection by itself should not be a contraindication for endovascular treatment in stroke patients with intracranial large vessel occlusion. Although more challenging endovascular procedures are to be suspected in both patients with CAD or ACAO, accurate distinction between CAD and ACAO might influence clinical decision making as better clinical outcome can be expected in patients with CAD.

## Introduction

In approximately a quarter of all patients with ischemic stroke due to large vessel occlusion (LVO), ipsilateral extracranial internal carotid artery (ICA) occlusion is accompanying the intracranial LVO. This phenomenon is known as tandem occlusion (1). Major causes of tandem occlusion are carotid artery dissection (CAD) and atherosclerotic carotid artery occlusion (ACAO) (2–4). During endovascular treatment (EVT) of ischemic stroke, the presence of an extracranial tandem occlusion may constitute a challenge to the neuro-interventionist as the obstructed internal carotid artery might hamper access to the intracranial target occlusion and potentially increases the risk of procedural complications of endovascular treatment (EVT). Furthermore, an additional reconstruction of a dissected ICA with a collapse of the true lumen is more challenging than a straightforward embolic intracranial occlusion.

A meta-analysis of individual patient data of five pivotal EVT trials showed similar treatment effects in patients with and without tandem lesions, although no stratification was performed for the type of tandem lesion (5). However, differences between CAD and ACAO with regard to clinical outcome after EVT have been suggested. A *post-hoc* analysis of the MR CLEAN trial regarding type of tandem lesion found a strong treatment effect in patients with CAD and no treatment effect in patients with ACAO despite the small number of included patients with CAD (6). On the other hand, in a recent observational study, no differences in outcomes were observed in EVT-treated patients with CAD compared to patients with severe atherosclerotic stenosis or occlusion although patients with CAD were younger and had less cardiovascular risk factors (7).

The evidence for a benefit of EVT in patients suffering from concomitant ipsilateral CAD is still scarce (8). On the one hand, good clinical outcome can be expected in patients with CAD due to the lower age and the lower prevalence of cardiovascular risk factors found in this patient group. On the other hand, it could be suggested that clinical outcome after EVT and interventional aspects in patients with CAD are comparable to outcomes in patients with ACAO as successful reperfusion rates may be lower and procedure times longer than in patients without a tandem lesion. Furthermore, CAD is known to be a source of emboli that can lead to re-occlusion or occlusion of a different vascular territory after initial successful recanalization. This may result in poorer clinical outcome in patients with successful reperfusion compared to patients without any extracranial carotid occlusion (9). The aim of this study was to assess neurological, functional and technical outcome, as well as safety, after EVT for intracranial LVO in patients with CAD. Comparisons will be made with patients with ACAO and patients without CAD or ACAO.

## Methods

### Patients

We studied patients who were included in the MR CLEAN trial or MR CLEAN Registry.

The multicenter randomized MR CLEAN trial consisted of 500 ischemic stroke patients with a NIHSS score of 2 points or more. After confirmation of a proximal intracranial occlusion of the anterior circulation on radiographic imaging, patients were randomized for EVT plus usual care (intervention group) or usual care only (control group) (10). For the current study, only patients randomized in the intervention group were included. Written informed consent before randomization was provided by all patients or their legal representatives. The study protocol was approved by a central medical ethics committee and the research board of each participating center.

The MR CLEAN Registry is an ongoing observational cohort study of consecutive patients with acute ischemic stroke undergoing EVT in the Netherlands (11). Enrollment of patients started directly after the last inclusion in the MR CLEAN trial on March 16, 2014. EVT consisted of mechanical thrombectomy and/or aspiration whether or not combined with arterial delivery of a thrombolytic agent. The exact choice of procedure was left to the discretion of the interventionist. Included patients in the current study were treated between March 16, 2014 and June 15, 2016. In order to create a study population comparable to the MR CLEAN trial, patients from the MR CLEAN Registry were included in our study when they were 18 years or older and had undergone EVT for acute ischemic stroke in one of the 16 MR CLEAN centers. The MR CLEAN Registry was approved by the ethics committee of the Erasmus University MC, Rotterdam, The Netherlands and permission to carry out the study as a registry was granted. With this approval it was approved by the research board of each participating center. At UMC Utrecht, approval for the study has been obtained from their own research board and ethics committee.

In our study, patients with CAD at the symptomatic side of ischemic stroke were compared to patients with ACAO to evaluate the relevance of distinguishing different type of extracranial carotid occlusions, and to patients without CAD or ACAO to evaluate the beneficial effects of EVT in patients with CAD.

### Imaging

Assessment of extracranial carotid artery on digital subtraction angiography (DSA) imaging was performed by an imaging core lab of neuro-interventionists who were unaware of clinical characteristics and clinical outcomes. CAD was assessed on computed tomography angiography (CTA) and digital subtraction angiography (DSA) and defined as follows: a double lumen contour with a narrow eccentric true lumen (string sign), periluminal hematoma and/or widening of the carotid artery (pseudo-aneurysm). The carotid bulb is spared and the dissection stops at the skull base. ACAO was defined as an occluded internal carotid artery with no contrast filling at the level of the carotid bulb with sign of atherosclerotic disease (calcifications). The possibility of a pseudo-occlusions caused by hampering of flow due to distal occlusions was excluded. Pseudo-occlusions were defined as a gradual decay in contrast filling in the ICA and a patent carotid bulb. Furthermore, the core lab evaluated the Alberta Stroke Program Early CT Score (ASPECTS) on baseline non-contrast CT (NCCT), occluded arterial segment and collateral grade score on baseline CTA, recanalization grades by the modified Thrombolysis In Cerebral Infarction (mTICI) on DSA, and presence of intracranial hemorrhage on follow up NCCT (12–14).

### Outcomes

Primary outcome was the change in stroke severity at short-term by comparing the National Institute of Health Stroke Scale (NIHSS) score at 24–48 h after treatment vs. baseline (delta-NIHSS). A negative delta-NIHSS indicates a neurological improvement while a positive value indicates a neurological decline at follow-up. Delta-NIHSS was chosen as primary outcome for the following reasons (a) lack of a control group of patients with LVO in whom EVT was not performed; and (b) baseline differences between the treatment groups which could result in frailer persons in one group with a subsequent effect on midterm functional outcomes. Secondary outcomes were NIHSS at 24 h, functional outcome at 3 months assessed with the modified Rankin Scale score (mRS), technical success parameters, and safety parameters.

Technical aspects of interventional procedure were evaluated regarding duration of procedure (groin puncture to closure of puncture site), recanalization status (mTICI score), and occurrence new clots in a different vascular territory. Complete DSA runs including anteroposterior and lateral views were required to reach a mTICI score of 2B or higher. Missing lateral views automatically resulted in a maximum possible mTICI score of 2A. Successful reperfusion was considered when mTICI 2B or higher was achieved. In addition, impact of reperfusion grade on functional outcome was studied.

Safety measures included progression of ischemic stroke and symptomatic intracranial hemorrhage (sICH). Ischemic stroke progression was defined as neurological deterioration of at least 4 points on the NIHSS, in which an intracranial hemorrhage was excluded as the cause of the deterioration with CT. ICH was considered symptomatic if the patient had died or had deteriorated neurologically (a decline of at least 4 points on the NIHSS), and the hemorrhage was related to the clinical deterioration (according to Heidelberg criteria). Both complications were evaluated by the complication committee consisting of two vascular neurologists and one neuroradiologist (15).

### Statistical Analysis

Baseline characteristics were presented as frequencies, mean with standard deviation or median and interquartile ranges (IQR). Intergroup differences were assessed with Fisher's exact test for categorical data and Mann-Whitney U test for continuous data. To evaluate the primary outcome, we assessed the neurological improvement by subtracting NIHHS at baseline from NIHSS at follow-up and tested within patient differences with a paired Wilcoxon signed rank test.

Neurological improvement in patients with CAD compared to patients with ACAO and to patient without CAD or ACAO was evaluated with linear regression analysis with NIHSS at follow up as outcome with adjustments for age, sex, NIHSS baseline, time from stroke onset to groin puncture, intravenous thrombolysis (IVT), location of intracranial occlusion, and collateral status. The functional outcome (mRS score) at long-term follow-up between the groups were expressed as an adjusted common odds ratio (acOR) with 95% confidence interval (95%CI) obtained from ordinal logistic regression with adjustments for age, sex, NIHSS baseline, time from stroke onset to groin puncture, intravenous thrombolysis (IVT), location of intracranial occlusion, and collateral status. For outcome regression analyses, multiple imputation techniques were used to provide adjusted, unbiased estimates (16, 17). All analyses were performed with R statistical software (version 3.4.2) and additional packages (rms, tableone, haven, Hmisc, ggplot, and ggpaired).

## Results

**Figure 1:**
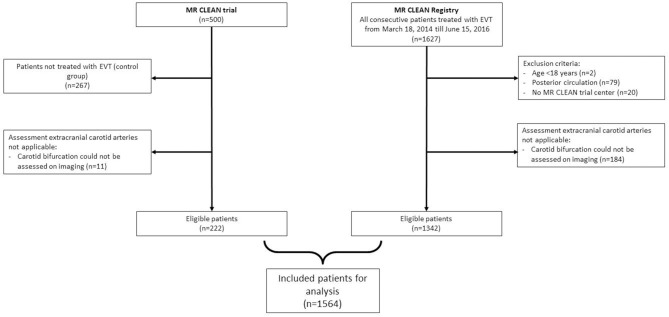
Flowchart of included patients for analysis.

Of the 233 patients who were allocated to intervention in the MR CLEAN trial, imaging assessment of the extracranial carotid artery was insufficient in 11 patients. In total, 222 patients of the MR CLEAN trial were included in our study. Of the 1,627 patients in the MR CLEAN Registry, 1,342 patients remained, after exclusion of those younger than 18 years of age, had an intracranial occlusion in the posterior circulation, were treated in a non-MR CLEAN trial center, or had insufficient imaging of the extracranial carotid artery. The combined dataset consisted of a total of 1,564 patients (Figure 1).

We identified 74 of 1,564 patients (4.7%) with CAD at the symptomatic side of ischemic stroke of which 15/233 (6.4%) patients in the MR CLEAN trial and 59/1,342 (4.4%) patients in the Registry. ACAO at the symptomatic side of stroke was observed in 92 /1,564 patients (5.9%), leaving 1,398/1,564 (89%) patients without CAD or ACAO.

### Baseline Patient Characteristics

Compared to patients with ACAO, patients with CAD were significantly younger and had less cardiovascular risk factors (Table 1). Furthermore, location of intracranial occlusion did not differ significantly. In both groups, most intracranial occlusions were found in the M1 segment of the middle cerebral artery (Table 1).

**Table 1:** Baseline characteristics of included patients.

	**Patients with CAD** **(*n* = 74)**	**Patients with ACAO** **(*n* = 92)**	**Patients without CAD or ACAO** **(*n* = 1398)**	***P*-value** **CAD vs. ACAO**	***P*-value** **CAD vs. without CAD or ACAO**
Sex-male (%)	55 (74.3)	72 (78.3)	727 (52.0)	0.69	<0.001
Age-years (median [IQR])	52 [45–59]	68 [58–77]	71 [60–80]	<0.001	<0.001
Smoking (%)	21 (28.4)	36 (39.6)	313 (22.5)	0.18	0.31
Diabetes mellitus (%)	2 (2.7)	15 (16.7)	228 (16.4)	0.01	0.01
Atrial fibrillation (%)	7 (9.6)	3 (3.3)	344 (24.9)	0.17	0.01
Hypertension (%)	18 (24.3)	38 (41.8)	704 (51.0)	0.03	<0.001
Myocardial infarction (%)	2 (2.7)	12 (13.2)	224 (16.3)	0.04	0.01
Previous stroke (%)	6 (8.3)	7 (7.6)	241 (17.3)	1.00	0.07
Hypocholesteremia (%)	5 (6.8)	19 (21.1)	418 (30.8)	0.02	<0.001
Pre-stroke independence (mRS ≤ 2) (%)	70 (95.9)	90 (98.9)	1221 (88.6)	0.46	0.08
Stroke severity at baseline (NIHSS) (median [IQR])^a^	16 [12–19]	16 [11–19]	16 [12– 20]	0.43	0.81
Location intracranial occlusion (%)				0.42	<0.001
Other	0 (0.0)	0 (0.0)	11 (0.8)	
Intracranial ICA	16 (22.2)	17 (18.7)	46 (3.4)	
ICA-T	25 (34.7)	33 (36.3)	298 (21.9)	
M1	26 (36.1)	39 (42.9)	828 (60.8)	
M2	5 (6.9)	2 (2.2)	178 (13.1)	
ASPECTS score at baseline (median [IQR])^b^	8 [7–10]	8 [7–9]	9 [7–10]	0.57	0.23
Collateral grading score (%)^c^				0.14	0.36
0	2 (2.9)	1 (1.1)	94 (7.0)	
1	28 (40.0)	27 (29.7)	435 (32.5)	
2	27 (38.6)	32 (35.2)	516 (38.5)	
3	13 (18.6)	31 (34.1)	294 (22.0)	
Treatment with intravenous thrombolysis (%)	63 (85.1)	79 (85.9)	1086 (77.7)	1.00	0.17
Duration from stroke onset to groin puncture (median [IQR])	235 [173–295]	211 [178–271]	210 [160–270]	0.54	0.15

a National Institute of Health Stroke scale (Scores range from 0 to 42, higher scores indicating severe stroke).

b Alberta Stroke Program Early Computed tomography Score (Scores range from 0 to 10 lower scores indicating more early ischemic changes on baseline NCCT).

c *Assessed at baseline CTA. A score of 0 indicated absent collateral supply to the occluded territory, 1: filling of >0% but ≤50%, 2: filling of >50% but <100%, 3: filling of 100% collateral supply of the occluded territory*.

Compared to patients without CAD or ACAO, patients with CAD were more frequently men (resp. 52% vs. 74%; *p* < 0.001), were younger (median age 71 years vs. 52 years; *p* < 0.001) and cardiovascular risk factors or history regarding diabetes mellitus, atrial fibrillation, hypertension, myocardial infarction, and hypercholesterolemia were less frequently present (Table 1). Median NIHSS scores at baseline were 16 in both groups (median 16 vs. 16 points; p 0.81). In both groups, the M1 segment of the middle cerebral artery was the most frequent location of the intracranial occlusion. Patients with CAD more frequently had a distal ICA or carotid terminus (ICA-T) occlusion, while patients without ACAO more often had an M2 occlusion (Table 1). On baseline imaging, patients with CAD had a lower ASPECT scores (median 8 points vs. 9 points; p 0.23) indicating more ischemic changes but this difference was not statistically significant. Duration from stroke onset to groin puncture (median 210 min vs. 235 min; p 0.15) and the administration of IVT (78% vs. 85%; p 0.17) were not statistically different (Table 1).

### Primary Outcome

**Figure 2:**
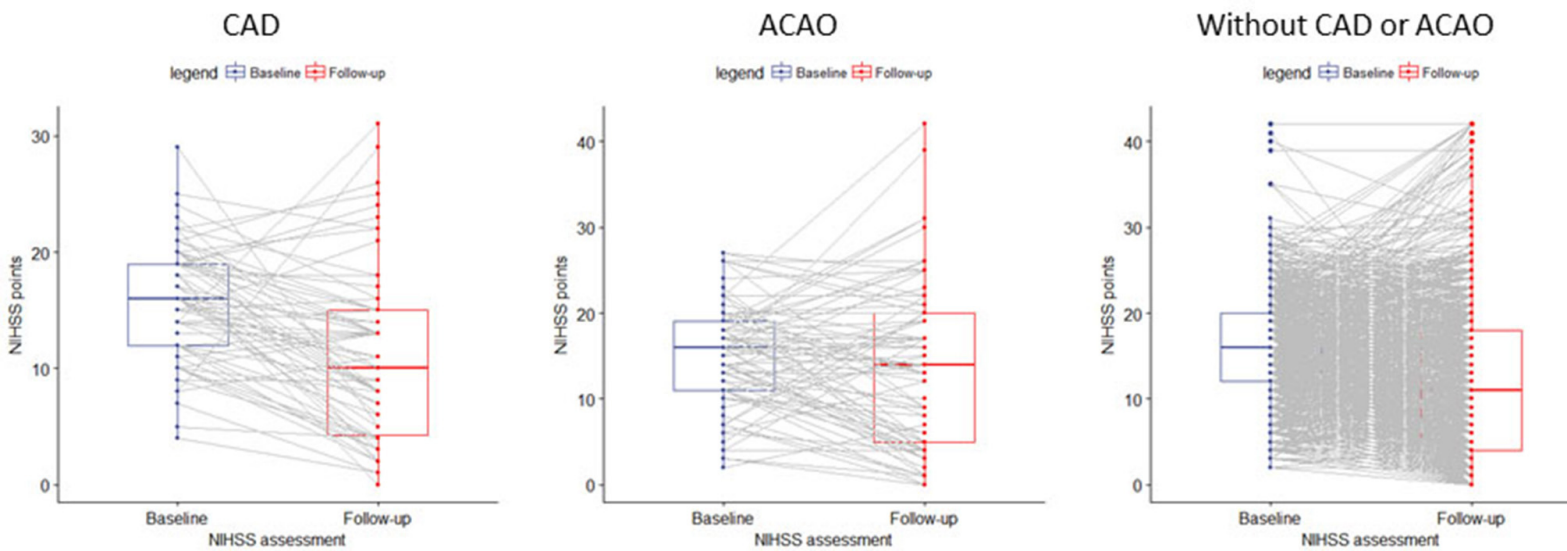
Paired boxplot of NIHSS points at baseline and follow-up.

A within-patient comparison showed a significant reduction in NIHSS in patients with CAD (NIHSS −5±7 points; *p* < 0.001), in patient with ACAO (NIHSS −1±9 points; *p* < 0.001) and in patients without CAD or ACAO (NIHSS −4±8 points; *p* < 0.001). In total, we observed a neurological improvement after EVT (delta-NIHSS < 0) in 50/70 (71%) patients with CAD, in 46/81 (57%) patients with ACAO and in 866/1,258 (69%) patients without CAD or ACAO (Figure 2).

### Outcomes in Patients With CAD vs. Patients With ACAO

**Figure 3:**
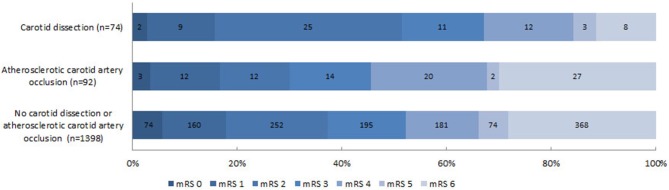
Distribution of the modified Rankin Scale (mRS) score at 90 days. Functional outcomes were statically significant different between patients with carotid artery dissection and with atherosclerotic carotid artery occlusion but also compared to patients without carotid artery dissection or atherosclerotic occlusion (resp. cOR 2.05; 95%CI 1.17–3.57 and cOR 1.60; 95%CI 1.07–2.38). After adjustments for sex, age, stroke severity (NIHSS) at baseline, time from stroke onset to intervention hospital, intravenous thrombolysis, collateral status and location of intracranial occlusion, functional outcome was no longer statistically different (resp. acOR 1.72; 95%CI 0.84–3.50 and acOR 1.00; 95%CI 0.64–1.56).

Compared to patients who had an extracranial tandem lesion due to ACAO, patients with CAD had a better neurological outcome on the NIHSS after adjustments for baseline parameters (NIHSS points −4.0 [95%CI −7.2–−0.8]) (Table 2). Functional outcome (mRS) was not significantly better in patients with CAD (acOR 1.72 [95% 0.84–3.50]) (Figure 3). Duration of procedure (resp. median 80 vs. 74 min; p 0.35) and rates of successful reperfusion (resp. 38/81 (47%) vs. 33/70 (47%); p 1.00) were comparable between both groups. In addition, we observed similar impact of reperfusion grade on functional outcome in patients with CAD and ACAO (Table 4).

**Table 2:** Clinical, technical and safety outcomes.

	**Patients with CAD** **(*n* = 74)**	**Patients with ACAO** **(*n* = 92)**	**Patients without CAD or ACAO** **(*n* = 1398)**	***P*-value** **CAD vs. ACAO**	***P*-value** **CAD vs. without CAD or ACAO**
**CLINICAL**					
Delta-NIHSS (mean [sd])	−5 (7)	−1 (9)	−4 (8)	0.03	0.62
NIHSS follow up (24−48 h) (median [IQR])	10 [4–15]	14 [5–20]	11 [4–18]	0.10	0.63
mRS score at 90 days (median [IQR])^a^	2 [2–4]	4[2–6]	3 [2–6]	0.01	0.01
mRS score at 0−2 at 90 days (%)	36 (51.4)	27 (30.0)	486 (37.3)	0.01	0.02
Mortality within 90 days (%)	8 (11.4)	27 (30.0)	368 (28.2)	0.01	0.01
**TECHNICAL**					
Duration procedure (median [IQR])	74 [51–99]	80 [57–115]	65 [40–90]	0.35	0.01
New thrombus different vascular territory – no. (%)	4 (22.2)	9 (39.1)	74 (25.6)	0.41	0.97
Successful recanalization (%)^b^	33 (47.1)	38 (46.9)	792 (58.4)	1.00	0.08
**SAFETY**					
Progression of ischemic stroke (%)	9 (12.2)	15 (16.3)	163 (11.7)	0.60	1.00
Symptomatic intracranial hemorrhage (%)^c^	4 (5.4)	10 (10.9)	83 (5.9)	0.33	1.00

a Modified Rankin scale score was assessed at 90 days after stroke onset.

b mTICI ≥ 2B, score of 0 indicates no perfusion or anterograde flow beyond occlusion site, 1: penetration of contrast but not perfusion, 2A: some perfusion <50% of vascular territory, 2B: substantial perfusion ≥50%, 3: complete perfusion of vascular territory.

c *Clinical deterioration due to intracranial hemorrhage defined by the Heidelberg bleeding classification*.

**Table 4:** Proportions of functional independence stratified by reperfusion grades.

	**Patients with CAD** **(*****n*** **=** **74)**		**Patient with ACAO** **(*****n*** **=** **92)**		**Patients without CAD or ACAO** **(*****n*** **=** **1,398)**	
mTICI-score^*^	*N* (%)	mRS 0–2 (%)	*N* (%)	mRS 0–2 (%)	*N* (%)	mRS 0–2 (%)
0–1	15	4 (19)	20	2 (10)	266	41 (15)
2a	19	11(58)	22	7 (32)	272	81 (30)
2b−3	32	19 (59)	47	15 (32)	729	348 (48)
cOR (95%CI)	1.35 (1.02–1.78)		1.34(1.18–1.66)		1.36(1.28–1.44)	

* *Score of 0 indicates no perfusion or anterograde flow beyond occlusion site, 1: penetration of contrast but not perfusion, 2A: some perfusion <50% of vascular territory, 2B: substantial perfusion ≥50, 3: complete perfusion of vascular territory*.

### Outcomes in Patients With CAD vs. Patients Without CAD or ACAO

Neurological improvement on the NIHSS at 24–48 h after EVT in patients with CAD and in patients without CAD or ACAO was similar (*p* = 0.62; Table 2). Also, the 24–48 h NIHSS scores in patients with CAD and patients without CAD or ACAO were similar after adjustment for baseline parameters (NIHSS points 0 [95%CI −2.1–2.0]).

Patients with CAD had a better functional outcome (mRS at 90 days) than patients without CAD or ACAO (cOR 1.60 [95%CI 1.07–2.38]). However, after adjustment for the prespecified prognostic factors, this association was no longer significant (acOR 1.00 [95%CI 0.64–1.56]) (Figure 2).The duration of procedure was significantly longer in patients with CAD than in patients without CAD or ACAO (median 74 vs. 65 min; p 0.01). Rate of successful reperfusion was significantly lower in patients with CAD (33/70 (47%) vs. 792/1357 (58%); p 0.08). Among patients with CAD, progression of ischemic stroke (resp. 12.2% vs. 11.7%; p 1.00) nor symptomatic intracranial hemorrhage (5% vs. 6%; p 1.00) occurred more often than in patients without CAD or ACAO. Impact of reperfusion grade on functional outcome was comparable between patients with CAD and patients without COD (Table 3).

**Table 3:** Univariable and multivariable analysis of functional outcome in EVT-treated patients with CAD.

	**Univariable**		**Multivariable**	
	**cOR**	**95%CI**	**acOR**	**95%CI**
Sex (male)	0.51	0.20 –1.30	0.49	0.18 –1.36
Age	0.95	0.92 –0.99	0.98	0.93 –1.02
Stroke severity (NIHSS) at baseline	0.90	0.82 –0.98	0.91	0.83 –1.01
Time from stroke onset to groin puncture	1.00	0.99 –1.01	1.00	0.99 –1.01
Intravenous thrombolysis	2.38	0.74 –7.67	3.39	0.94 –12.19
Collateral status	2.17	1.20 –3.90	2.11	1.02 –4.36
Location of intracranial occlusion	1.09	0.66 –1.79	0.81	0.47 –1.39

*cOR, common odds ratio; acOR, adjusted common odds ratio; 95%CI, 95% confidence interval*.

### Determinants of Functional Outcome in Patients With CAD

Age (cOR 0.95 [95%CI 0.92–0.99]), baseline NIHSS (cOR 0.90 [95%CI 0.83–0.98]) and collateral status on CTA (cOR 2.20 [95%CI 1.13–4.29]) were significantly associated with a good functional outcome in patients with CAD. (Table 3). However, in multivariable analysis, only collateral status on CTA remained independently associated with good functional outcome (acOR 2.11 [95%CI 1.02–4.36]) (Table 3).

## Discussion

In this observational study, neurological improvement after EVT in patients with CAD was significantly larger than in patients with a tandem occlusion due to ACAO, while rates of successful reperfusion were similar. Short term neurological improvement after EVT between patients with CAD and those without CAD or ACAO was of similar magnitude. However, at 3 months follow-up, patients with CAD were significantly more often functionally independent compared than patients without CAD or ACAO. This can be explained by the fact that patients with CAD were younger and had less cardiovascular risk factors. These findings are consistent with a recent systematic review that showed that a large proportion of patients with tandem occlusion due to CAD achieved functional independency after EVT (18). Furthermore, we observed longer procedural times and lower rates of successful reperfusion in patients with CAD and ACAO. This might reflect the technical challenge of passing the extracranial carotid artery obstruction.

Regarding safety aspects, no differences were found between EVT-treated patients with CAD or without CAD/ACAO. Although passing the intimal tear in a CAD carries the risk of dislodging new emboli, embolization of thrombus to a new vascular territory did not occur more often in the CAD group. Furthermore, the rate of symptomatic ICH was not higher in patients with CAD, despite our finding that CAD patients suffered from larger occlusions (more distal ICA and ICA-T occlusions) and had a lower ASPECTS score at baseline. Our finding that the rate of complications in EVT-treated patients with CAD is not increased, corresponds with previous research (18, 19).

In a recent observational study, patients with CAD were compared to patients with severe stenosis (>90%) or occlusion due to atherosclerotic disease (7). Procedural times were comparable, however, rates of successful reperfusion were lower in our study compared to other cohort studies on endovascular treatment in clinical practice (20, 21). This finding might be explained by our core laboratory assessment which reduces overestimation due to operator bias (22, 23). Also, multiple retrospective studies on EVT-treated patients with CAD, showed comparable results regarding outcomes and safety aspects (19, 24, 25).

Several limitations of our study need to be addressed. Firstly, we could not evaluate the effectiveness of EVT due to the absence of an untreated control group in our observational study. A pooled analysis of patient-level data of larger EVT trails would be possible, however, only two trials (MR CLEAN and REVASCAT) allowed for inclusion of patients with CAD (10, 26). Performing a randomized controlled trial would be best to determine whether EVT is effective compared to no EVT. This may not be feasible in light of the low number of patients with an intracranial occlusion in combination with a CAD. Furthermore, despite extracranial tandem lesions, intracranial occlusions are nowadays routinely treated in daily practice based on previous results (5, 27). Our results confirm that this is a reasonable policy. Secondly, we performed an observational multicenter study. Selection of patients and treatment strategy may vary between intervention centers, introducing selection bias. We tried to minimize this bias by adjusting for prognostic factors in multivariable analyses, and by comparing different groups of patients. Thirdly, due to the retrospective design of our study, we were not able to perform additional analyses on the technical management of tandem lesions. This data would be of interest as there is an ongoing debate whether a diseased ICA should be treated or left alone during EVT. In addition, recent literature, suggests that the treatment of the intracranial occlusion should have priority over possible treatment of the affected ICA (28–31). Fourthly, the long-term follow-up was no longer than 3 months in the MR CLEAN Registry. Therefore, we were not able to assess the occurrence of recurrent strokes over a longer time period.

We observed that EVT in patients with ipsilateral CAD are technically more challenging due to the additional obstruction of extracranial carotid artery. However, neurological improvement at short term is not different from patients without CAD or ACAO and suggests a comparable treatment effect. At follow-up, the chances of gaining functional independence are even larger in patients with CAD compared to patients without CAD or ACAO, likely because patients with CAD are younger, healthier, and are burdened less with atherosclerotic and cardiac disease.

In summary, EVT in patients with intracranial LVO due to CAD results in neurological improvement comparable to patients without tandem occlusion. Therefore, carotid artery dissection by itself should not be a contraindication for endovascular treatment in stroke patients with intracranial large vessel occlusion. Although more challenging endovascular procedures are to be suspected in both patients with CAD or ACAO, accurate distinction between CAD and ACAO might influence clinical decision making as better clinical outcome can be expected in patients with CAD.

## Ethics Statement

The MR CLEAN trial was carried out in accordance with the recommendations of Medical Ethics Committee and Resarch Board of Erasmus MC University Medical Center with written informed consent from all subjects. All subjects gave written informed consent in accordance with the Declaration of Helsinki. The protocol was approved by the ethics committee of the Erasmus MC, Rotterdam, the Netherlands (MEC-2014-235). For the MR CLEAN Registry, the central medical ethics committee of the Erasmus Medical Center Rotterdam, the Netherlands, evaluated the study protocol and granted permission to carry out the study as a registry.

## Author Contributions

KC contributed to the study design, drafting the manuscript and figures, and performed statistical analysis. RG, PvD, and BE contributed to the data acquisition and drafting the manuscript. MU, RvO, and WvZ contributed to the study design and drafting the manuscript. DD and AvdL contributed to the conception and study design, interpretation of data, and drafting the manuscript. AvE contributed to the conception and the study design, contributed to the data acquisition, and drafting the manuscript. All authors contributed to manuscript revision, read and approved the submitted version.

### Conflict of Interest Statement

The authors declare that the research was conducted in the absence of any commercial or financial relationships that could be construed as a potential conflict of interest.
