# Supplementary material for: Acute Endovascular Treatment of Patients With Ischemic Stroke From Intracranial Large Vessel Occlusion and Extracranial Carotid Dissection
Source: Front Neurol. 2019 Feb 19;10:102. doi: 10.3389/fneur.2019.00102 (PMC6390807; doi:10.3389/fneur.2019.00102)
Supplement: Supplementary file 1 [file Data_Sheet_1.docx]

MR CLEAN Investigators – group authors

Executive committee

Diederik W.J. Dippel^1^;Aad van der Lugt^2^;Charles B.L.M. Majoie^3^;Yvo B.W.E.M. Roos^4^;Robert J. van Oostenbrugge^5^;Wim H. van Zwam^6^;Jelis Boiten^14^;Jan Albert Vos^8^

Study coordinators

Ivo G.H. Jansen^3^; Maxim J.H.L. Mulder^1,2^;Robert- Jan B. Goldhoorn^5,6^;Kars C.J. Compagne^2^;Manon Kappelhof^3^

Local principal investigators

Wouter J. Schonewille^7^;Jan Albert Vos^8^;Charles B.L.M. Majoie^3^;Jonathan M. Coutinho^4^;Marieke J.H. Wermer^9^;Marianne A.A. van Walderveen^10^;Julie Staals^5^;Wim H. van Zwam^6^;Jeannette Hofmeijer^11^;Jasper M. Martens^12^;Geert J. Lycklama à Nijeholt^13^;Jelis Boiten^14^;Bob Roozenbeek^1^;Bart J. Emmer^2^;Sebastiaan F. de Bruijn^15^;Lukas C. van Dijk^16^;H. Bart van der Worp^17^ ;Rob H. Lo^18^;Ewoud J. van Dijk^19^;Hieronymus D. Boogaarts^20^;Paul L.M. de Kort^21^;Jo J.P. Peluso^26^;Jan S.P. van den Berg^22^;Boudewijn A.A.M. van Hasselt^23^;Leo A.M. Aerden^24^;René J. Dallinga^25^;Maarten Uyttenboogaart^28^;Omid Eshghi^29^;Tobien H.C.M.L. Schreuder^30^;Roel J.J. Heijboer^31^;Koos Keizer^32^;Lonneke S.F. Yo^33^;Heleen M. den Hertog^22^;Emiel J.C. Sturm^35^

Imaging assessment committee

Charles B.L.M. Majoie^3^(chair);Wim H. van Zwam^6^;Aad van der Lugt^2^;Geert J. Lycklama à Nijeholt^13^;Marianne A.A. van Walderveen^10^;Marieke E.S. Sprengers^3^;Sjoerd F.M. Jenniskens^27^;René van den Berg^3^;Albert J. Yoo^37^;Ludo F.M. Beenen^3^;Alida A. Postma^6^;Stefan D. Roosendaal^3^;Bas F.W. van der Kallen^13^;Ido R. van den Wijngaard^13^;Adriaan C.G.M. van Es^2^;Bart J. Emmer^2,3^;Jasper M. Martens^12^; Lonneke S.F. Yo^33^;Jan Albert Vos^8^; Joost Bot^36^, Pieter-Jan van Doormaal^2^.

Writing committee

Diederik W.J. Dippel^1^(chair);Aad van der Lugt^2^;Charles B.L.M. Majoie^3^;Yvo B.W.E.M. Roos^4^;Robert J. van Oostenbrugge^5^;Wim H. van Zwam^6^;Geert J. Lycklama à Nijeholt^13^;Jelis Boiten^14^;Jan Albert Vos^8^;Wouter J. Schonewille^7^;Jeannette Hofmeijer^11^;Jasper M. Martens^12^;H. Bart van der Worp^17^;Rob H. Lo^18^

Adverse event committee

Robert J. van Oostenbrugge^5^(chair);Jeannette Hofmeijer^11^;H. Zwenneke Flach^23^

Trial methodologist

Hester F. Lingsma^38^

Research nurses / local trial coordinators

Naziha el Ghannouti^1^;Martin Sterrenberg^1^;Corina Puppels^7^;Wilma Pellikaan^7^;Rita Sprengers^4^;Marjan Elfrink^11^;Joke de Meris^14^;Tamara Vermeulen^14^;Annet Geerlings^19^;Gina van Vemde^22^;Tiny Simons^30^;Cathelijn van Rijswijk^21^;Gert Messchendorp^28^;Hester Bongenaar^32^;Karin Bodde^24^;Sandra Kleijn^34^;Jasmijn Lodico^34^; Hanneke Droste^34^;M. Wollaert^5^;D. Jeurrissen^5^;Ernas Bos^9^;Yvonne Drabbe^15^;Nicoline Aaldering^11^;Berber Zweedijk^17^;Mostafa Khalilzada^15^.

PhD / Medical students:

Esmee Venema^38^; Vicky Chalos^1,38^; Ralph R. Geuskens^3^; Tim van Straaten^19^; Saliha Ergezen^1^; Roger R.M. Harmsma^1^; Daan Muijres^1^; Anouk de Jong^1^; Wouter Hinseveld^7^;Olvert A. Berkhemer^1,3,6^;Anna M.M. Boers^3,39^; J. Huguet^3^;P.F.C. Groot^3^;Marieke A. Mens^3^;Katinka R. van Kranendonk^3^;Kilian M. Treurniet^3^;Manon L. Tolhuijsen^3^;Heitor Alves^3^.

List of affiliations

Department of Neurology^1^, Radiology^2^, Public Health^38^, Erasmus MC University Medical Center;

Department of Radiology and Nuclear Medicine^3^, Neurology^4^, Biomedical Engineering & Physics^39^, Amsterdam UMC, University of Amsterdam, Amsterdam;

Department of Neurology^5^, Radiology^6^, Maastricht University Medical Center and Cardiovascular Research Institute Maastricht (CARIM);

Department of Neurology^7^, Radiology^8^, Sint Antonius Hospital, Nieuwegein;

Department of Neurology^9^, Radiology^10^, Leiden University Medical Center;

Department of Neurology^11^, Radiology^12^, Rijnstate Hospital, Arnhem;

Department of Radiology^13^, Neurology^14^, Haaglanden MC, the Hague;

Department of Neurology^15^, Radiology^16^, HAGA Hospital, the Hague;

Department of Neurology^17^, Radiology^18^, University Medical Center Utrecht;

Department of Neurology^19^, Neurosurgery^20^, Radiology^27^, Radboud University Medical Center, Nijmegen;

Department of Neurology^21^, Radiology^26^, Sint Elisabeth Hospital, Tilburg;

Department of Neurology^22^, Radiology^23^, Isala Klinieken, Zwolle;

Department of Neurology^24^, Radiology^25^, Reinier de Graaf Gasthuis, Delft;

Department of Neurology^28^, Radiology^29^, University Medical Center Groningen;

Department of Neurology^30^, Radiology^31^, Atrium Medical Center, Heerlen;

Department of Neurology^32^, Radiology^33^, Catharina Hospital, Eindhoven;

Department of Neurology^34^, Radiology^35^, Medical Spectrum Twente, Enschede;

Department of Radiology^36^, Amsterdam UMC, Vrije Universiteit van Amsterdam, Amsterdam;

Department of Radiology^37^, Texas Stroke Institute, Texas, United States of America.
